# FIRM‐P separates knee phenotype from patient context in anterior cruciate ligament‐related instability: A conceptual framework integrating arthrogenic muscle inhibition as a cross‐domain modifier

**DOI:** 10.1002/jeo2.70914

**Published:** 2026-09-19

**Authors:** Dario Giunchi, Matteo Denti, Marc Barrera Uso

**Affiliations:** ^1^ Ars Medica Clinic Gravesano Switzerland; ^2^ Department of Orthopaedic Surgery and Traumatology HFR Fribourg – Cantonal Hospital Fribourg Switzerland

**Keywords:** anterior cruciate ligament, arthrogenic muscle inhibition, clinical decision‐making, knee instability, knee phenotype, return to sport

## Abstract

**Purpose:**

Clinical reasoning after anterior cruciate ligament (ACL) injury is still often built on isolated proxies such as imaging, instrumented laxity, functional testing or patient‐reported instability. These variables are clinically useful, but they are not interchangeable and do not answer the same question. The purpose of this work is to propose FIRM‐P (Functional, Individual, Radiological and Mechanical instability plus patient context) as a practical conceptual framework that separates the ACL‐related knee phenotype from patient‐context indication, while integrating arthrogenic muscle inhibition (AMI) as a cross‐domain modifier rather than as a separate instability pillar.

**Methods:**

FIRM‐P has two layers. FIRM describes the knee through Functional, Individual, Radiological and Mechanical instability. P describes the patient context through performance demands, priorities and expectations, rehabilitation potential and personal modifiers. Each domain is interpreted as favourable, borderline or unfavourable. Discordant patterns do not directly produce treatment decisions; they trigger a verification loop. AMI is integrated as a mechanism that may alter the expression of Functional and Individual instability, may coexist with true ACL insufficiency and may influence the timing of elective surgery.

**Results:**

After verification, an explicit classification rule assigns the knee to one of four mutually exclusive phenotypes: low‐instability/non‐ACL, occult or associated‐instability, compensated ACL‐deficient or overt ACL‐instability. Patient context is applied only after the knee has been phenotyped. A simplified decision pathway is provided in the main manuscript; the full operational 32‐scenario matrix and an interactive HTML prototype that allows readers to simulate the algorithm across the 32 operational scenarios are provided as Supporting Information.

**Conclusions:**

FIRM‐P is proposed as a conceptual framework, not a validated score. Its immediate value is to organize ACL‐related instability reasoning into a transparent sequence: phenotype the knee, verify discordance and then apply patient context. Its future value will depend on content validation, reliability testing and prospective derivation work.

**Level of Evidence:**

Level V.

## Graphical abstract

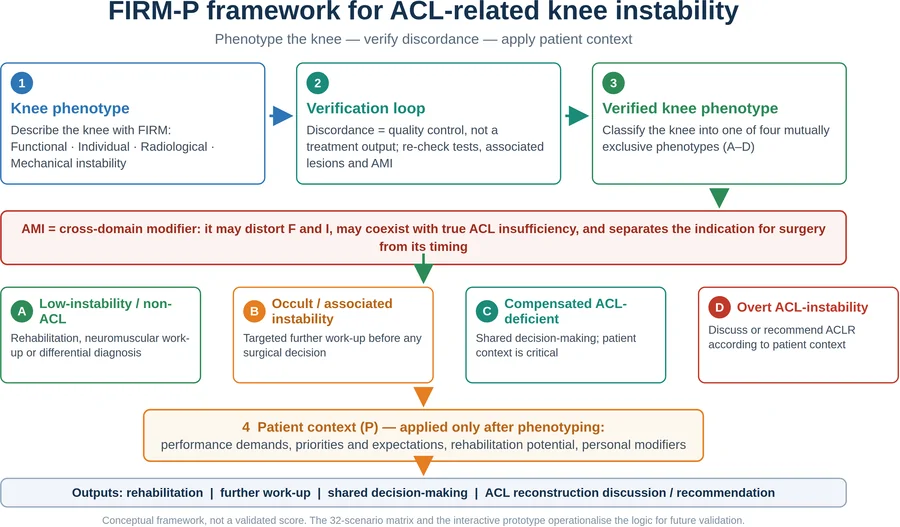

**Abbreviations**
ACL: anterior cruciate ligament
ACLR: anterior cruciate ligament reconstruction
ALL: anterolateral ligament
AMI: arthrogenic muscle inhibition
EMG: electromyography
FIRM: Functional, Individual, Radiological and Mechanical instability
FIRM‐P: Functional, Individual, Radiological and Mechanical instability plus patient context
IKDC: International Knee Documentation Committee
KT‐1000/KT‐2000: knee arthrometers
LESS: Landing Error Scoring System
MAT: Movement Analysis Test
MRI: magnetic resonance imaging
P: patient context
PROM: patient‐reported outcome measure
RTS: return to sport
SANTI: Scientific Anterior Cruciate Ligament Network International
VMO: vastus medialis obliquus

## INTRODUCTION

Anterior cruciate ligament (ACL)‐related clinical reasoning is still frequently reduced to isolated proxies. Magnetic resonance imaging (MRI), instrumented laxity, return‐to‐sport (RTS) tests and patient‐reported instability are often discussed as if they described the same construct, yet they do not. This matters because a knee may look structurally abnormal, behave well, feel unstable or be mechanically lax without these findings fully overlapping [1, 10, 15].

A second problem is that knee‐level information and patient‐level indication are routinely conflated. A knee may deserve reconstruction biologically or mechanically, but the patient may not need that level of stability for everyday life. Conversely, a highly demanding patient may seek maximal stability even when the verified knee phenotype does not strongly support ACL insufficiency. Decision‐making studies and consensus documents increasingly support this separation between the knee problem and the patient problem [6, 12].

A third problem is that ACL injury is not only a structural event. Arthrogenic muscle inhibition (AMI), also described in the Scientific Anterior Cruciate Ligament Network International (SANTI) work as centrally derived motor inhibition after knee injury or surgery, can produce quadriceps activation failure, hamstring overactivity, active extension deficit, degraded movement quality and loss of confidence in the limb. AMI can therefore mimic, amplify or temporally distort both functional instability and perceived instability. If it is ignored, clinicians risk over‐interpreting some findings and under‐interpreting others [9, 21, 24, 25].

The purpose of this paper is to propose FIRM‐P (Functional, Individual, Radiological and Mechanical instability plus patient context) as a clinically practical and scientifically disciplined conceptual model. The framework is designed to answer three questions in sequence: What does the knee show? Are the findings internally coherent? What do those findings mean for this patient?

## THE FRAMEWORK IN THREE CLINICAL QUESTIONS

**Figure 1:**
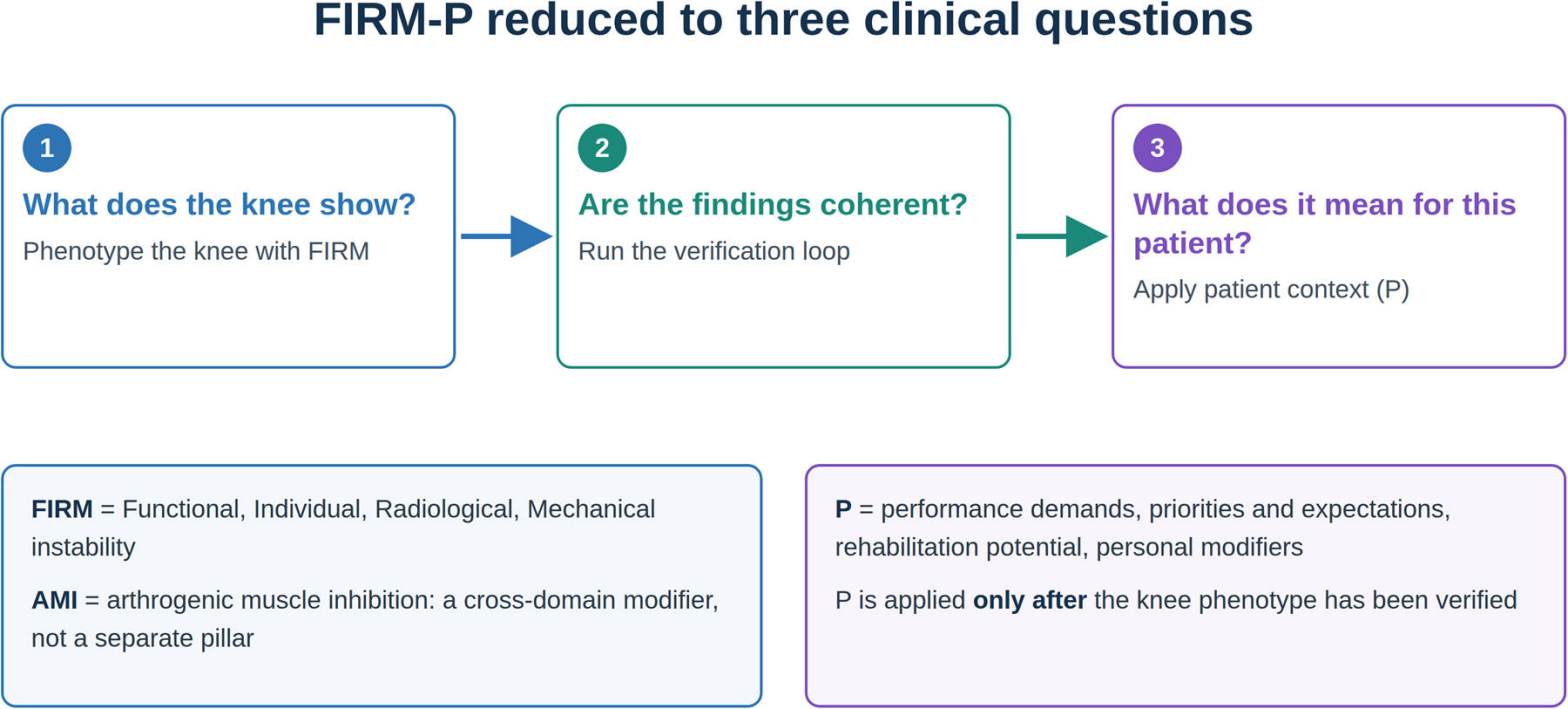
FIRM‐P reduced to three clinical questions. The framework first describes the knee, then tests the coherence of the findings, and only afterwards applies patient context.

The central idea of FIRM‐P is simple. The knee should be described first, the coherence of the findings should be tested second, and the patient should be contextualized only afterwards. This sequence prevents the framework from collapsing into either a purely structural model or a purely demand‐based model (Figure 1).

In practice, the framework is best understood as a layered system. FIRM describes the knee‐instability phenotype. The verification loop determines whether the current pattern is trustworthy as measured. P then determines the practical importance of the verified knee phenotype for that specific patient.

## THE FIRM‐P DOMAINS

For conceptual robustness, each domain is better treated as ordinal than binary. In other words, domains are best described as favourable, borderline or unfavourable rather than as simply positive or negative. Binary simplification may still be useful in operational algorithms, but the conceptual framework should acknowledge the reality of clinical grey zones (Table 1).

**Table 1:** The FIRM‐P domains at a glance.

Component	Core question	Typical inputs	What it mainly adds	What it cannot do alone
F—Functional instability	How does the injured limb behave in motion?	RTS laboratory testing, force plates, movement analysis, gait analysis, video, wearables, hop tests with quality metrics	Whole‐limb dynamic control and movement quality	It cannot prove structural insufficiency by itself
I—Individual instability	How unstable does the knee feel to the patient?	Giving‐way history, confidence, activity‐specific instability, instability‐focused PROM content	The lived instability experience	It can be influenced by fear, compensation or AMI
R—Radiological instability profile	What does imaging reveal about the structural environment?	ACL/graft appearance, menisci, peripheral ligaments, ALL, slope, notch morphology, associated lesions	Structural context and imaging‐supported risk modifiers	It cannot substitute for mechanical or functional expression
M—Mechanical instability	Is objective laxity present?	KT‐1000/KT‐2000 or equivalent, stress radiography such as Telos	Quantifiable mechanical support	It does not describe function, symptoms or patient demands
P—Patient context	How important is stability for this patient?	Sport/occupation, expectations, rehabilitation capacity, modifiers	Clinical meaning of the verified knee phenotype	It should not override an unverified knee phenotype

Abbreviations: ACL, anterior cruciate ligament; ALL, anterolateral ligament; AMI, arthrogenic muscle inhibition; FIRM‐P, Functional, Individual, Radiological and Mechanical instability plus patient context; KT‐1000/KT‐2000, knee arthrometers; PROM, patient‐reported outcome measure; RTS, return to sport.

## FUNCTIONAL INSTABILITY

Functional instability is defined here as an instrumentally demonstrable dynamic control deficit of the injured lower limb. This is broader than hop distance and broader than the knee seen in isolation. It includes movement quality, loading symmetry, timing, limb coordination and behaviour during gait, landing, deceleration, directional change and RTS tasks [10, 15].

This definition intentionally shifts the focus from the ACL as an isolated structure toward the ACL‐deficient limb as a dynamic system. In clinical practice, this is the domain most likely to detect subtle dynamic instability, maladaptive compensation, or poor rotational control that may remain partly invisible on static imaging or incompletely represented by simple laxity testing.

This tiered interpretation is consistent with prior RTS work by Gokeler, Welling, Zaffagnini and colleagues, who proposed a multicomponent test battery combining strength testing, hop performance, jump‐landing quality, International Knee Documentation Committee (IKDC) and ACL‐Return to Sport after Injury (RSI) to support RTS decision‐making, and with subsequent prospective work showing that a substantial proportion of patients do not meet comprehensive RTS criteria even 9 months after anterior cruciate ligament reconstruction (ACLR) (Table 2) [11, 29].

**Table 2:** Tiered operationalisation of the functional domain.

Tier	Description	Examples	Practical role
Tier 1	High‐resource laboratory biomechanics	3D motion capture, force plates, instrumented RTS batteries, landing/deceleration/cutting analysis, gait protocols	Preferred reference when available
Tier 2	Clinic‐based instrumented movement analysis	MAT/Green Room‐type systems, clinic‐based force platforms, KneeKG‐like gait analysis, synchronized video plus force platform	Bridge between research biomechanics and daily practice
Tier 3	Scalable surrogate functional assessment	2D video analysis, LESS‐type assessments, wearable sensors/IMUs, hop tests interpreted with movement quality	Broad clinical applicability and follow‐up

Abbreviations: 2D, two‐dimensional; 3D, three‐dimensional; IMUs, inertial measurement units; LESS, Landing Error Scoring System; MAT, Movement Analysis Test; PROM, patient‐reported outcome measure; RTS, return to sport.

Movement Analysis Test (MAT) with Green Room and knee kinesiography with KneeKG‐type systems fit within the clinic‐based Tier 2 space. MAT is described as a movement‐quality assessment using high‐speed cameras, a force platform and six standardized movements. KneeKG‐like systems provide in‐load gait‐based information about tibiofemoral behaviour during treadmill walking [8]. Within FIRM‐P, these tools are valuable because they populate the Functional domain; they are not universal gold standards, and they are not stand‐alone surgical triggers [7, 13, 16].

## INDIVIDUAL INSTABILITY

Individual instability captures the patient's lived experience of instability. It includes giving‐way episodes, lack of trust, avoidance behaviour and instability during daily life or sport‐specific tasks. It should be assessed using targeted interview content and, where relevant, instability‐related patient‐reported outcome measure items. It is not a generic satisfaction domain. AMI matters here because a patient may experience insecurity or inability to trust the limb even when structural or mechanical findings are limited, especially in the early phase after injury or surgery [14, 21, 24].

## RADIOLOGICAL INSTABILITY PROFILE

The radiological domain is intentionally broader than ‘MRI positive or negative’. It should be understood as a radiological instability profile that includes ACL or graft morphology, meniscal lesions, peripheral ligament injury, anterolateral structures when relevant and anatomical risk modifiers such as posterior tibial slope or notch morphology. This broader definition prevents imaging from becoming a simplistic yes/no judgement on the ACL alone and allows it to describe the structural environment that may support, challenge or complicate the interpretation of instability. Associated lesions such as ramp lesions and lateral meniscus posterior root tears deserve specific attention because they may be difficult to detect on MRI and may contribute to anteroposterior or rotational instability [3, 4, 19].

## MECHANICAL INSTABILITY

Mechanical instability captures objective, quantifiable laxity. In practical terms, it is populated by arthrometric methods such as KT‐1000/KT‐2000 or equivalent devices and by stress radiography such as Telos‐based protocols. This is the most directly quantifiable domain in the framework, but it still does not replace function, perception or imaging. The role of M is to provide mechanical support, not to summarize the whole clinical problem [17].

## PATIENT CONTEXT

Patient context answers a different question from FIRM. FIRM asks what the knee shows. P asks what that verified knee phenotype means for this patient. In practical terms, P should be described through four subdomains: performance demands, priorities and expectations, potential for rehabilitation and personal modifiers. The most useful clinical simplification is often low‐demand versus high‐demand context, but the framework should not reduce patient context to sport alone. Occupational demands, willingness to modify activity, ability to complete rehabilitation, age in context and global risk‐benefit balance all matter [6, 12].

## AMI AS A CROSS‐DOMAIN MODIFIER

AMI should be integrated into the framework, but it should not become a separate sixth pillar. The reason is conceptual. Functional instability, patient‐experienced instability, imaging and mechanical laxity are descriptive domains. AMI is not another parallel descriptor of the same type. It is a biological and neurophysiological mechanism that can alter how instability is expressed across domains.

Using the international standard term AMI, and acknowledging the broader centrally derived motor inhibition work described by Sonnery‐Cottet and colleagues, the key message is the same: after knee injury or surgery, altered afferent input, pain, effusion, haemarthrosis and neuroplastic changes can lead to quadriceps activation failure and often to hamstring overactivity. Clinically, this may produce active extension deficit, degraded whole‐limb control and a powerful sense that the knee is not functioning normally [18, 20, 21, 22, 24].

AMI therefore plays three roles in FIRM‐P. First, it is a mechanistic explanation for some discordant patterns, especially functional and/or subjective abnormalities with limited structural or mechanical support. Second, it can coexist with true ACL insufficiency, meaning that the knee may be both structurally unstable and secondarily inhibited. Third, it is a timing modifier because unresolved AMI increases the perioperative risk environment and should be addressed before elective ligament surgery whenever possible (Table 3) [21, 23, 24, 25, 26].

**Table 3:** AMI as a cross‐domain modifier within FIRM‐P.

AMI‐related feature	Typical clinical expression	Domains most affected	Interpretive implication
Quadriceps/VMO inhibition	Poor quadriceps recruitment, inability to actively extend well, reduced patellar lift	Mainly F and I	Functional or subjective abnormality may be amplified without equivalent R or M abnormality
Hamstring overactivity and extension deficit	Antalgic flexion posture, reversible extension loss in prone fatigue manoeuvres	F, I and timing of surgery	The clinician should distinguish neuromotor inhibition from a fixed mechanical block
Pain, effusion, haemarthrosis, associated acute injury	Early painful knee, swelling, reduced confidence, protective co‐contraction	All domains indirectly, especially F and I	Supports the AMI safeguard within the verification loop
Reversible Grades (1a/2a) versus refractory Grades (1b/2b) versus chronic Grade 3	Temporary inhibition, persistent inhibition or chronic fixed extension deficit	Interpretation and timing	AMI is not all‐or‐none; reversibility and chronicity matter clinically

Abbreviations: AMI, arthrogenic muscle inhibition; FIRM‐P, Functional, Individual, Radiological and Mechanical instability plus patient context; VMO, vastus medialis obliquus.

Clinically, one useful distinction is between a truly locked knee caused by a displaced intra‐articular structure and a pseudo‐locked knee driven by hamstring contracture and quadriceps inhibition. The SANTI technical note is relevant here because it shows that a proportion of extension deficits after knee injury or surgery can be rapidly reversed when the clinician specifically addresses hamstring contracture, haemarthrosis and quadriceps/vastus medialis obliquus (VMO) activation failure [5]. Emerging evidence also suggests that electromyographic biofeedback may improve knee extension and balance during rehabilitation after ACLR, although the evidence base remains limited [2].

Practical AMI screening signals should remain simple: pain, effusion or haemarthrosis, obvious quadriceps or VMO inhibition, active extension deficit, hamstring contracture and an early post‐injury or early postoperative context should all raise suspicion. In the Sonnery‐Cottet/SANTI grading logic, Grade 0 corresponds to normal VMO contraction; Grades 1a and 1b to isolated quadriceps/VMO activation failure, reversible with simple exercises or refractory to them; Grades 2a and 2b to inhibition with hamstring contracture and extension deficit, again reversible or refractory; and Grade 3 to a chronic fixed extension deficit that is irreducible without extensive posterior arthrolysis. This grading system is not the focus of the present paper, but it is relevant because it shows that AMI has clinically important stages and is not a minor technical curiosity [22, 23, 24, 25].

## THE VERIFICATION LOOP

A core rule of the framework is that discordant patterns are not treatment outputs. They are quality‐control alerts. The verification loop exists to avoid false certainty, not to force artificial concordance. In practice, it means re‐checking test quality, acquisition technique, interpretation, overlooked associated lesions, and the possibility that active AMI is distorting what the clinician is seeing (Table 4).

**Table 4:** Verification loop for discordant FIRM patterns.

Discordant pattern	What should be re‐checked	Most likely explanations
F and/or I unfavourable while R and M are favourable	Imaging quality, laxity acquisition, adequacy of the functional test, presence of AMI	Neuromuscular deficits, proprioceptive deficits, AMI, non‐ACL explanations
R favourable but M unfavourable	Mechanical testing technique, MRI quality, possible static‐imaging limitations	False‐negative imaging, elongation, structurally insufficient but poorly visualized ACL/graft state
R unfavourable but M favourable	Clinical relevance of imaging and quality of mechanical testing	Associated lesions, rotational pathology, imaging not translating into anterior laxity
R and/or M unfavourable while F remains favourable	Task difficulty and whole‐limb assessment strategy	Compensated ACL deficiency, coper behaviour, non‐decompensated knee
F unfavourable while I remains favourable	How instability was questioned and whether AMI is active	Poor awareness, compensation, early AMI, insufficiently demanding questioning
I unfavourable while F remains favourable	Task selection and reproducibility of instability in function	Intermittent instability, psychological amplification, early AMI‐related insecurity

Abbreviations: ACL, anterior cruciate ligament; AMI, arthrogenic muscle inhibition; FIRM, Functional, Individual, Radiological and Mechanical instability; MRI, magnetic resonance imaging.

### AMI safeguard

If clinically relevant AMI is active, it should beaddressed before finalizing elective surgical timing unless another urgent lesion takes precedence.This safeguard does not mean that AMI excludes ACL insufficiency. It means that AMI can distort F and I, can coexist with true structural instability and can make the timing of surgery less safe if ignored [5, 23, 26, 27].

## VERIFIED KNEE PHENOTYPES

Once discordance has been processed through the verification loop, the knee can be classified into one of four practical phenotypes (Table 5). The purpose of these phenotypes is not to replace clinical judgement but to simplify the transition from complex data to coherent reasoning.

**Table 5:** Verified knee phenotypes after the verification loop.

Phenotype	Definition (after verification)	Default implication
A. Low‐instability/non‐ACL phenotype	R and M favourable, regardless of the state of F and I.	Favour rehabilitation, neuromuscular work‐up or differential diagnosis rather than ACLR; actively consider AMI when F and/or I are abnormal.
B. Occult or associated‐instability phenotype	R unfavourable with M favourable: structural abnormality without mechanical confirmation.	Favour targeted further work‐up for associated intra‐articular or rotational pathology before offering surgery or dismissing instability.
C. Compensated ACL‐deficient phenotype	M unfavourable (with or without R unfavourable), with F and/or I relatively preserved.	Recognize coper or compensated behaviour; patient context becomes critical.
D. Overt ACL‐instability phenotype	M unfavourable with both F and I unfavourable (with or without R unfavourable).	This is the clearest branch for offering or recommending ACLR depending on patient context.

Abbreviations: ACL, anterior cruciate ligament; ACLR, anterior cruciate ligament reconstruction; AMI, arthrogenic muscle inhibition.

To keep the four phenotypes mutually exclusive and exhaustive, an explicit classification rule is applied after verification. Verified mechanical instability is the discriminating objective domain. When M is unfavourable, the knee is classified as ACL‐insufficient: the phenotype is overt ACL‐instability (D) when both clinical expressions of instability (F and I) are also unfavourable and compensated ACL‐deficient (C) otherwise. When M is favourable, an unfavourable radiological profile defines the occult or associated‐instability phenotype (B), and a favourable radiological profile defines the low‐instability/non‐ACL phenotype (A), whatever the state of F and I. Under this rule, each of the 16 possible FIRM patterns maps to exactly one phenotype, and the same verified pattern always yields the same knee phenotype irrespective of patient context; patient context modifies only the downstream management stance (Data S1).

## PRACTICAL DECISION PATHWAY

**Figure 2:**
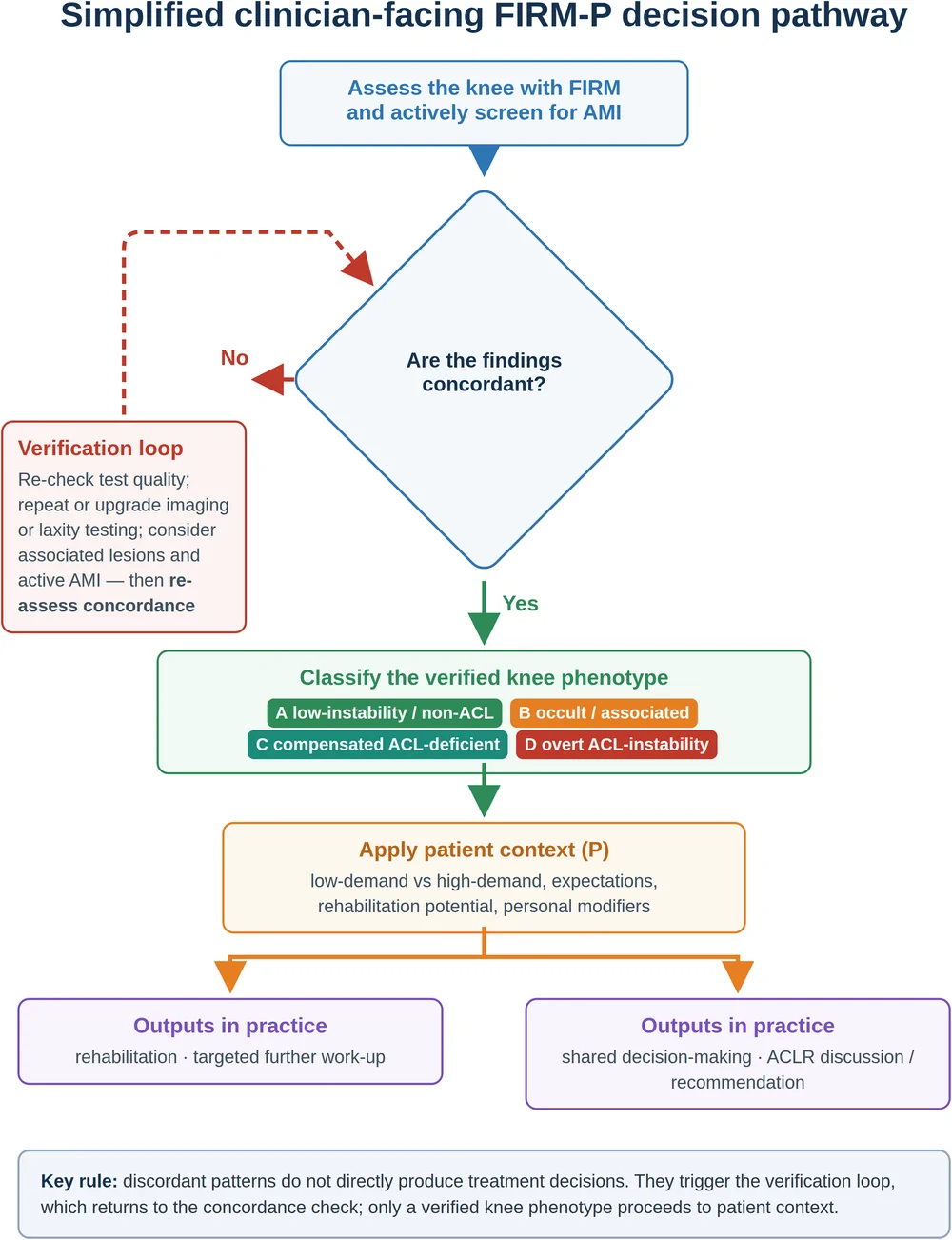
Simplified clinician‐facing FIRM‐P decision pathway. The verification loop returns to the concordance check; only a verified knee phenotype proceeds to patient context. ACL, anterior cruciate ligament; ACLR, anterior cruciate ligament reconstruction; AMI, arthrogenic muscle inhibition; FIRM‐P, Functional, Individual, Radiological and Mechanical instability plus patient context.

The practical use of the framework can be reduced to five steps (Figure 2). First, assess the knee with FIRM and actively screen for AMI. Second, decide whether the pattern is concordant or discordant. Third, if discordant, enter the verification loop rather than choosing treatment immediately. Fourth, classify the verified knee phenotype. Fifth, apply patient context to determine whether the correct output is rehabilitation, further work‐up, shared decision‐making or ACLR.

If R and M remain favourable after verification, ACLR should not be proposed purely because F and/or I are abnormal. This is the branch most likely to reflect AMI or other non‐ACL neuromuscular drivers.

Isolated radiological abnormality is not enough. Imaging can identify important pathology, but without mechanical or convincing functional support it should not alone drive ACLR.

Isolated mechanical abnormality deserves respect because it is objective, but in low‐demand patients it should usually trigger confirmation and shared decision‐making rather than automatic reconstruction.

In high‐demand patients, confirmed mechanical abnormality lowers the threshold for recommending ACLR, particularly when combined with any additional unfavourable domain.

A functional abnormality alone, including one detected by MAT‐, KneeKG‐ or force‐plate‐like tools, should trigger better understanding of the limb, not automatic surgery.

Whenever AMI is active, the clinician should separate the indication for surgery from the timing of surgery. A knee may ultimately merit reconstruction, but surgery should not be timed through uncontrolled AMI unless urgency overrides that principle.

For transparency, the full 32‐scenario operational matrix accompanies the manuscript as Data S1. The conceptual framework itself remains ordinal; the appendix is a pragmatic binary translation of the same logic for case‐by‐case use. Data S2 provides an interactive HTML prototype of the FIRM‐P Decision Navigator, which simulates the same 32 scenarios and returns the corresponding phenotype, verification requirement, immediate action and surgical stance. The prototype is included only as a practical demonstration of the reasoning pathway and is not proposed as a validated medical device, prediction model or stand‐alone treatment recommendation tool. A separate visual abstract summarizes the framework for rapid reader orientation.

## CLINICAL VIGNETTES

The following hypothetical vignettes illustrate how FIRM‐P can be applied in daily practice. They are not patient data and are not intended to validate the framework; they demonstrate the sequence of reasoning: assess FIRM, verify discordance, address AMI when relevant, classify the knee phenotype and then apply patient context. The four vignettes correspond to the four verified knee phenotypes (A–D) (Table 6).

**Table 6:** Hypothetical clinical vignettes demonstrating practical use of the FIRM‐P sequence, one for each verified knee phenotype.

Vignette	Initial FIRM‐P pattern	Reasoning through the framework	Practical output
1. AMI‐dominant apparent instability (Phenotype A)	F unfavourable, I unfavourable, R favourable, M favourable, P‐high, AMI active/suspected.	The initial pattern is discordant because functional and perceived instability are not supported by imaging or objective laxity. The verification loop should re‐check imaging and mechanical testing, but AMI, strength deficit, proprioceptive deficit and kinetic‐chain dysfunction become the leading explanations if R and M remain favourable.	ACLR should not be proposed unless new hard evidence emerges. AMI‐directed treatment, neuromuscular rehabilitation and reassessment are prioritized.
2. Occult or associated instability (Phenotype B)	F favourable, I unfavourable (activity‐specific giving‐way), R unfavourable (suspected ramp lesion), M favourable, P‐high, no active AMI.	Perceived instability is supported by the radiological profile but not by instrumented laxity. The verification loop targets the quality of mechanical testing and a dedicated imaging review, because ramp lesions and lateral meniscus posterior root tears may escape standard MRI reading and may produce rotational rather than anteroposterior laxity.	Targeted further work‐up (dedicated imaging review and, when justified, examination under anaesthesia or arthroscopic evaluation) before any decision about reconstruction; confirmed associated lesions are treated on their own merits.
3. Compensated ACL‐deficient high‐demand knee (Phenotype C)	F favourable, I favourable, R unfavourable, M unfavourable, P‐high, no active AMI.	The knee may be mechanically and structurally abnormal while still functionally compensated. After confirmation of R and M and exclusion of testing error, the phenotype is best interpreted as compensated ACL‐deficient. Patient context becomes decisive because high rotational demands make future instability clinically relevant despite preserved function at the time of assessment.	ACLR may be recommended/proposed after confirmation and shared decision‐making. The same verified knee phenotype in a P‐low context would more naturally lead to discussion rather than automatic recommendation.
4. Overt instability with active AMI (Phenotype D)	F unfavourable, I unfavourable, R unfavourable, M unfavourable, P‐high, AMI Grade 2a 3 weeks after injury.	The pattern is concordant and the phenotype is overt ACL‐instability, so the indication question is clear. The AMI safeguard, however, separates the indication for surgery from its timing: Hamstring contracture and quadriceps activation failure should be addressed first, because operating through uncontrolled AMI worsens the perioperative risk environment.	ACLR is recommended in this high‐demand context; elective surgery is scheduled after the extension deficit resolves and quadriceps activation recovers, unless another urgent lesion overrides this principle.

Abbreviations: ACL, anterior cruciate ligament; ACLR, anterior cruciate ligament reconstruction; AMI, arthrogenic muscle inhibition; FIRM‐P, Functional, Individual, Radiological and Mechanical instability plus patient context; MRI, magnetic resonance imaging.

## DISCUSSION

The main contribution of FIRM‐P is conceptual clarification. The framework separates the instability phenotype of the knee from the clinical importance of that phenotype for the patient. This sounds simple, but it addresses a common source of confusion in ACL practice: moving directly from an imaging, laxity or functional finding to a treatment decision without first establishing whether the overall pattern is coherent. The explicit classification rule guarantees that the same verified FIRM pattern always produces the same knee phenotype and confines patient context to the management layer [1, 6, 10, 12, 15].

The second contribution is that FIRM‐P preserves the non‐interchangeability of the major domains. Function, patient‐experienced instability, imaging and mechanical laxity are all clinically relevant, but they do not carry the same information. The available ACL literature supports this multidomain view rather than a single‐test approach [1, 10, 15].

The third contribution is the integration of AMI without unnecessary proliferation of pillars. AMI is important precisely because it explains why some early functional and subjective presentations look unstable even when the structural/mechanical domains are limited, and why some structurally unstable knees may perform surprisingly well. Treating AMI as a cross‐domain modifier preserves conceptual clarity and reflects the available pathophysiological and clinical data better than creating a redundant extra domain [9, 20, 21, 24].

The framework also has important limitations. First, it is conceptual and has not yet undergone content validation, reliability testing, derivation or external validation. Second, the operational 32‐scenario matrix in Data S1 is a pragmatic binary translation of a fundamentally ordinal framework and will require refinement in future consensus work; in particular, the classification rule privileges verified mechanical laxity as the discriminating objective domain, a deliberate and conservative choice that consensus work may refine. Third, Tier 2 and Tier 3 functional tools are clinically valuable but heterogeneous in construct coverage and standardization. Fourth, the exact quantitative relationship between AMI, functional instability and patient‐experienced instability remains plausible and well reasoned but not yet formally modelled in prospective validation studies.

These limitations do not weaken the purpose of the paper; they define it. The role of the present manuscript is to provide a durable conceptual architecture on which later consensus, reliability and validation studies can be built. The framework should therefore be read as a disciplined reasoning model rather than as a finished score.

## FUTURE DIRECTIONS

A coherent validation pathway follows naturally from this conceptual paper. The next step should be a content‐validation and consensus process to define admissible measures for each domain, clarify favourable/borderline/unfavourable thresholds and refine discordant branches. That work should be followed by feasibility and inter‐rater reliability studies, and only then by prospective derivation and validation of any formal score or prediction model [28].

## CONCLUSION

FIRM‐P is proposed as a practical conceptual framework for ACL‐related knee instability. Its central proposal is simple: phenotype the knee first, verify discordance second and apply patient context only afterwards. The framework remains multidimensional without becoming conceptually diffuse because AMI is integrated as a cross‐domain modifier rather than as a separate pillar. Its immediate value is improved clinical reasoning; its long‐term value will depend on formal validation work.

## AUTHOR CONTRIBUTIONS

The authors contributed to the conception of the FIRM‐P framework, development of the initial clinical logic, conceptual refinement, clinical interpretation, critical revision and approval of the final manuscript.

## FUNDING INFORMATION

The authors have no funding to report.

## CONFLICT OF INTEREST STATEMENT

The authors declare no conflicts of interest. In particular, the authors have no financial or commercial relationship with the providers of the assessment systems mentioned in this manuscript.

## ETHICS STATEMENT

This manuscript presents a conceptual clinical framework and does not report individual patient data, new clinical datasets or research involving human participants or animals. No individual patient data are reported; the clinical vignettes are hypothetical.

## Supporting information

Supporting file 1: Supplementary Appendix A (32‐scenario operational matrix).

Supporting file 2: Interactive HTML prototype (Supplementary Digital Material 1).

## Data Availability

No datasets were generated or analysed for the present conceptual manuscript. The operational scenario matrix is provided as Data S1 and the interactive prototype is provided as Data S2.
