# Supplementary material for: FIRM‐P separates knee phenotype from patient context in anterior cruciate ligament‐related instability: A conceptual framework integrating arthrogenic muscle inhibition as a cross‐domain modifier
Source: J Exp Orthop. 2026 Sep 19;13(3):e70914. doi: 10.1002/jeo2.70914 (PMC13589612; doi:10.1002/jeo2.70914)
Supplement: Supplementary file 1 [file JEO2-13-e70914-s002.docx]

**Supplementary Appendix A**

**Operational 32-scenario matrix for the FIRM-P framework**

This appendix preserves the full case-by-case clinical logic of the FIRM-P framework. It should be read as an operational simplification of the broader ordinal model used in the main manuscript. In the tables below, + means unfavourable/pathological and - means favourable/non-pathological. P-low corresponds to low-demand or low-need-for-stability patients; P-high corresponds to high-demand patients who require maximal rotational stability.

Phenotype classification rule. Each verified FIRM pattern maps to exactly one knee phenotype, irrespective of patient context. When M is unfavourable, the knee is classified as ACL-insufficient: overt ACL-instability (D) when both F and I are unfavourable, compensated ACL-deficient (C) otherwise. When M is favourable, an unfavourable radiological profile defines the occult or associated-instability phenotype (B), and a favourable radiological profile defines the low-instability/non-ACL phenotype (A), whatever the state of F and I. Patient context (P-low vs P-high) modifies only the immediate action and the surgery stance, never the knee phenotype.

Appendix-wide AMI safeguard: when clinically relevant AMI is active, optimise it before final elective surgical timing unless urgency overrides this principle. AMI can distort Functional and Individual domains, can coexist with true ACL insufficiency, and should not be mistaken for a stand-alone surgical indication or exclusion criterion.

*Table A1. P-low scenarios (L01-L16).*

| **ID / FIRM-P** | **Phenotype** | **Immediate action** | **Surgery stance** |
| --- | --- | --- | --- |
| L01  P: P-low  F + \| I + \| R + \| M + | D \| Concordant overt ACL-instability phenotype in a low-demand patient. | Confirm the complete pattern and perform shared decision-making. | Offer and discuss ACL reconstruction; do not impose treatment. |
| L02  P: P-low  F + \| I + \| R + \| M - | B \| Clinical-functional-radiological pattern suggests pathology, but mechanical instability is not demonstrated. | Verification loop focused on M; if M remains negative, search for associated intra-articular or rotational lesions. | Do not trigger ACL reconstruction automatically; reconsider only if mechanical support emerges or ACL insufficiency is otherwise confirmed. |
| L03  P: P-low  F + \| I + \| R - \| M + | D \| Functional, perceived and mechanical pattern with MRI-negative imaging. | Verification loop focused on R; consider false-negative imaging or non-visualised insufficiency. | Offer and discuss ACL reconstruction after confirmation. |
| L04  P: P-low  F + \| I + \| R - \| M - | A \| Functional and perceived instability without hard support from imaging or laxity. | Verification loop: re-check R and M; consider repeating MRI or instrumented laxity if acquisition quality was suboptimal. Consider AMI / centrally derived motor inhibition as a potential explanation for functional and/or perceived instability without hard structural support. | No ACL reconstruction unless new hard evidence emerges. Prioritise targeted treatment of AMI if clinically present. |
| L05  P: P-low  F + \| I - \| R + \| M + | C \| Functional, radiological and mechanical evidence with little/no perceived instability. | Confirm the pattern and recognise possible compensation. | Offer and discuss ACL reconstruction after confirmation. |
| L06  P: P-low  F + \| I - \| R + \| M - | B \| Functional plus radiological abnormality without perceived or mechanical confirmation. | Verification loop focused on M and on the relevance of the radiological finding. | No automatic ACL reconstruction; if M remains negative, consider associated lesions or rotational dysfunction. |
| L07  P: P-low  F + \| I - \| R - \| M + | C \| Functional plus mechanical abnormality with no perceived instability and no radiological support. | Verification loop focused on R; consider occult insufficiency, elongation or compensated/coper behaviour. | Offer/discuss ACL reconstruction after confirmation; do not impose treatment. |
| L08  P: P-low  F + \| I - \| R - \| M - | A \| Isolated functional abnormality with no subjective, radiological or mechanical support. | Verification loop: re-check R and M; if confirmed negative, investigate neuromuscular control, strength, proprioception and kinetic-chain deficits. Consider AMI / centrally derived motor inhibition as a potential explanation for functional and/or perceived instability without hard structural support. | No ACL reconstruction if R and M remain negative. Prioritise targeted treatment of AMI if clinically present. |
| L09  P: P-low  F - \| I + \| R + \| M + | C \| Radiological and mechanical evidence with perceived instability, but preserved functional control. | Confirm the hard evidence and recognise possible coper behaviour. | Offer and discuss ACL reconstruction; low-demand profile prevents an automatic recommendation. |
| L10  P: P-low  F - \| I + \| R + \| M - | B \| Perceived instability plus radiological abnormality, but functional and mechanical testing are negative. | Verification loop focused on F and M; if discrepancy persists, investigate associated intra-articular or rotational lesions. | Do not trigger ACL reconstruction automatically unless hard support emerges. |
| L11  P: P-low  F - \| I + \| R - \| M + | C \| Perceived instability plus mechanical evidence without radiological confirmation. | Verification loop focused on R and on the quality of M acquisition; consider false-negative imaging or static MRI limitations. | Consider and discuss ACL reconstruction after confirmation; avoid automatic recommendation in a low-demand patient. |
| L12  P: P-low  F - \| I + \| R - \| M - | A \| Perceived instability without functional, radiological or mechanical support. | Verification loop focused on R and M; if they remain negative, consider non-ACL explanations and symptom interpretation. Consider AMI / centrally derived motor inhibition as a potential explanation for functional and/or perceived instability without hard structural support. | No ACL reconstruction if hard evidence remains absent. Prioritise targeted treatment of AMI if clinically present. |
| L13  P: P-low  F - \| I - \| R + \| M + | C \| Radiological plus mechanical evidence despite absent functional/perceived instability. | Re-check both R and M and assess for compensated/coper behaviour. | Offer and discuss ACL reconstruction after confirmation and shared decision-making. |
| L14  P: P-low  F - \| I - \| R + \| M - | B \| Radiological abnormality without functional, perceived or mechanical expression. | Verification loop focused on R; confirm that the radiological finding is truly relevant. | No ACL reconstruction if isolated R positivity is confirmed and all other domains remain favourable. |
| L15  P: P-low  F - \| I - \| R - \| M + | C \| Isolated mechanical abnormality in a low-demand patient. | Verification loop focused on R; consider false-negative MRI/static imaging and elongation phenomena. | Consider and discuss ACL reconstruction only after confirmation of persistent mechanical abnormality; avoid automatic indication. |
| L16  P: P-low  F - \| I - \| R - \| M - | A \| No evidence of ACL-related instability across FIRM in a low-demand patient. | No verification loop beyond routine quality control. | No ACL reconstruction. |

*Table A2. P-high scenarios (H01-H16).*

| **ID / FIRM-P** | **Phenotype** | **Immediate action** | **Surgery stance** |
| --- | --- | --- | --- |
| H01  P: P-high  F + \| I + \| R + \| M + | D \| Concordant overt ACL-instability phenotype in a high-demand patient. | No further verification beyond routine confirmation. | Recommend/propose ACL reconstruction. |
| H02  P: P-high  F + \| I + \| R + \| M - | B \| Functional, perceived and radiological pattern suggests pathology, but mechanical instability is not demonstrated. | Verification loop focused on M; if still negative, investigate associated lesions or rotational instability such as peripheral structures. | Propose ACL reconstruction only if mechanical support emerges or ACL insufficiency becomes clearly established. |
| H03  P: P-high  F + \| I + \| R - \| M + | D \| Functional, perceived and mechanical evidence with MRI-negative imaging. | Verification loop focused on R; consider false-negative imaging or non-visualised insufficiency. | Recommend/propose ACL reconstruction after confirmation. |
| H04  P: P-high  F + \| I + \| R - \| M - | A \| Functional and perceived instability without hard support from imaging or laxity. | Verification loop: re-check R and M; if needed, repeat imaging or instrumented laxity with better technique/quality. Consider AMI / centrally derived motor inhibition as a potential explanation for functional and/or perceived instability without hard structural support. | No ACL reconstruction unless hard evidence emerges; intensive rehabilitation is indicated. Prioritise targeted treatment of AMI if clinically present. |
| H05  P: P-high  F + \| I - \| R + \| M + | C \| Functional, radiological and mechanical evidence with little/no perceived instability. | Confirm the pattern and recognise possible compensation or high tolerance. | Recommend/propose ACL reconstruction. |
| H06  P: P-high  F + \| I - \| R + \| M - | B \| Functional plus radiological abnormality without perceived or mechanical confirmation. | Verification loop focused on M; if M remains negative, investigate associated lesions or rotational dysfunction before surgery. | No automatic ACL reconstruction; aggressive further work-up is justified in high-demand settings. |
| H07  P: P-high  F + \| I - \| R - \| M + | C \| Functional plus mechanical abnormality with high demands, but radiology is negative. | Verification loop focused on R; consider static imaging false negatives or elongation. | Recommend/propose ACL reconstruction after confirmation. |
| H08  P: P-high  F + \| I - \| R - \| M - | A \| Isolated functional abnormality in a high-demand patient. | Verification loop: re-check R and M; if confirmed negative, address neuromuscular control, strength, proprioception and sport-specific mechanics. Consider AMI / centrally derived motor inhibition as a potential explanation for functional and/or perceived instability without hard structural support. | No ACL reconstruction if R and M remain negative. Prioritise targeted treatment of AMI if clinically present. |
| H09  P: P-high  F - \| I + \| R + \| M + | C \| Radiological and mechanical evidence with perceived instability despite preserved functional control. | Confirm the hard evidence; recognise possible coper behaviour but high demands matter. | Recommend/propose ACL reconstruction. |
| H10  P: P-high  F - \| I + \| R + \| M - | B \| Perceived instability plus radiological abnormality, but mechanical instability is not demonstrated. | Verification loop focused on M; if M becomes positive, move toward ACL reconstruction. If M remains negative, investigate associated intra-articular or rotational causes. | No immediate ACL reconstruction without confirmation; low threshold for further work-up. |
| H11  P: P-high  F - \| I + \| R - \| M + | C \| Perceived instability plus mechanical evidence without radiological confirmation. | Verification loop focused on R and M quality; consider false-negative imaging or static MRI limitations. | Recommend/propose ACL reconstruction after confirmation. |
| H12  P: P-high  F - \| I + \| R - \| M - | A \| Perceived instability without functional, radiological or mechanical support. | Verification loop focused on R and M; if they remain negative, address neuromuscular and interpretive causes. Consider AMI / centrally derived motor inhibition as a potential explanation for functional and/or perceived instability without hard structural support. | No ACL reconstruction if hard evidence remains absent. Prioritise targeted treatment of AMI if clinically present. |
| H13  P: P-high  F - \| I - \| R + \| M + | C \| Radiological plus mechanical evidence despite absent symptoms and preserved function. | Confirm R and M, assess for compensated/coper behaviour. | Recommend/propose ACL reconstruction. |
| H14  P: P-high  F - \| I - \| R + \| M - | B \| Radiological abnormality without functional, perceived or mechanical confirmation in a high-demand patient. | Verification loop focused on M; if M remains negative, do not infer ACL insufficiency from R alone. | No ACL reconstruction if M stays negative. |
| H15  P: P-high  F - \| I - \| R - \| M + | C \| Isolated mechanical abnormality in a high-demand patient. | Confirm M and review imaging for false-negative findings or elongation. | Recommend/propose ACL reconstruction after confirmation. |
| H16  P: P-high  F - \| I - \| R - \| M - | A \| No evidence of ACL-related instability across FIRM despite high demands. | No verification loop beyond routine quality control. | No ACL reconstruction. |

Supplementary Digital Material 1. The FIRM-P Decision Navigator HTML prototype accompanies this appendix as a separate supplementary digital file. It allows readers to simulate each of the 32 scenarios and obtain the corresponding phenotype, immediate action, surgery stance, verification-loop message, and AMI safeguard. The prototype is included for educational and conceptual demonstration only and is not a validated score, medical device, or stand-alone treatment recommendation tool.
