# Supplementary material for: FIRM‐P separates knee phenotype from patient context in anterior cruciate ligament‐related instability: A conceptual framework integrating arthrogenic muscle inhibition as a cross‐domain modifier
Source: J Exp Orthop. 2026 Sep 19;13(3):e70914. doi: 10.1002/jeo2.70914 (PMC13589612; doi:10.1002/jeo2.70914)
Supplement: Supplementary file 2 [file JEO2-13-e70914-s001.html]

FIRM-P Clinical Simulator — Supplementary Digital Material 1

FIRM-P Clinical Simulator


FIRM-P · Decision support after ACL injury

# Your patient has a torn ACL. *Decide what to do — by reasoning, not opinion.*

**FIRM-P** — Giunchi, Denti & Barrera Uso · *Journal of Experimental Orthopaedics* (ESSKA)

Open framework — contributions & validation partners welcome

A peer-reviewed framework for physiotherapists and physicians: a shared assessment architecture — four measured domains, one explicit rule, verification of discordance, and only then the patient's context — supporting the treating physician in a defensible treatment decision. Every step tells you **what to do**, and shows you **why**.

**This is not a score.** Nothing is summed, nothing is weighted, no probability comes out — the domains are questions, not points, and the output is a reasoning sequence, not a number.

4+1

domains

1

explicit rule

32

scenarios

31

verified references

▶  See it work — 15 seconds
Try it yourself

Simulator32-scenario mapObjectionsEvidenceAuthorship

**Where it all starts**

An MRI report reads *«ACL tear»* — and the same argument begins, everywhere in the world: **operate**, say the surgeons; **rehabilitate**, say the physiotherapists. Both sides defend the patient. And both are arguing about one word — *«instability»* — as if it named one single thing.

**Why FIRM-P exists**

It doesn't. The devil is in the details: «unstable» is **four different, measurable questions** — F how the limb moves, I how the knee feels, R what imaging shows, M what laxity measures.

Measure each, integrate them, verify their disagreement — and the dispute dissolves into reasoning: **the treatment that fits this knee and this life**.

1

**Describe the knee**four domains: function, perception, imaging, laxity

2

**Verify discordance**disagreement is a quality-control alert, not a decision

3

**Then — the patient**context shapes management, never the diagnosis

“FIRM-P does not replace clinical judgement — it disciplines it. The decision stops being an opinion and becomes a measurement, verified, then reasoned together with the patient. The framework is conceptual and awaits formal validation: we publish it to be tested, criticised and improved.”

Dario GiunchiiD · Matteo DentiiD · Marc Barrera UsoiD — the authors

**This is an open framework.** Criticism, refinement, teaching and independent validation projects are not just permitted — they are the point. Authorship stays with the authors; progress belongs to everyone, and its benefit to the patient.

Contribute — write to the authors
studio.giunchi@hin.ch

Knee pattern: **0/4**
Reset
Copy summary
Share
Quiz mode
Copied ✓

STEP 1

F

### Functional instability [7–8, 10–16, 29]

Is there an instrumentally demonstrable dynamic control deficit? What counts? ↓

**Inputs** · instrumented RTS batteries, force plates, hop tests with quality metrics; clinic-based movement analysis such as MAT / Green Room-type systems [13] and KneeKG-type knee kinesiography [7, 8, 16]

Assessment checklist — structure your look, then answer

Hop-test battery below symmetry or movement-quality criteria
 Movement-quality deficit on landing, deceleration or cutting
 Gait abnormality under load
 Instrumented movement analysis abnormal (MAT / KneeKG-type)

A guide, not a calculator: any clinically relevant, reproducible deficit → consider answering Yes. The judgement stays with you, and ticked items are included in Copy summary.

Nodynamic control preservedfavourableYesmovement-quality / control deficitunfavourable

STEP 2

I

### Individual instability [1, 12]

Does the patient experience the knee as unstable?

**Inputs** · giving-way history, activity-specific instability, instability-focused PROM items

Nono relevant perceived instabilityfavourableYesgiving-way / lack of trustunfavourable

STEP 3

R

### Radiological instability profile [3, 4, 19]

Does imaging show a relevant structural instability profile?

**Inputs** · ACL/graft morphology, ramp lesions, lateral root tears, slope & notch morphology

Assessment checklist — structure your look, then answer

ACL / graft discontinuity or abnormal morphology
 Ramp lesion — posteromedial meniscocapsular junction
 Lateral meniscus posterior root tear
 Markedly increased posterior tibial slope
 Notch morphology / other anatomical risk modifiers

A guide, not a calculator: any clinically relevant finding → consider answering Yes. The judgement stays with you, and ticked items are included in Copy summary.

Nono relevant structural profilefavourableYesrelevant imaging findingsunfavourable

STEP 4

M

### Mechanical instability [17]

Is objective laxity demonstrated? **The discriminating domain.**

**Inputs** · KT-1000/KT-2000 arthrometry, Telos stress radiography, side-to-side difference; emerging: quantitative pivot-shift instrumentation (KiRA-type) [30, 31]

Nono objective laxityfavourableYesobjective laxity confirmedunfavourable

STEP 5

P

### Patient context [6, 12]

How much rotational stability does this patient actually need?

**Inputs** · pivoting sport exposure, occupational demand, psychological readiness (e.g. ACL-RSI), kinesiophobia, realistic expectations, rehabilitation potential

Low demanddaily life, linear activitiesHigh demandpivoting sport, heavy occupation

MODIFIER

!

### AMI screen [23–27]

Is arthrogenic muscle inhibition clinically active?

**Inputs** · quadriceps/VMO activation failure, extension deficit, hamstring contracture (SANTI grading)

Nonot evident — normal VMO activationYesactive / suspected — activation failure or extension deficit

Or load a worked case — one click

AMI-dominant pattern
Occult / associated lesion
Compensated ACL-deficient
Overt instability + AMI

Result ↓

## The whole matrix, as one map

Each cell is one F·I·R·M pattern; the colour is the verified knee phenotype. The colours are identical across the two panels — **patient context changes the management stance, never the phenotype**. Click any cell to load it.

### P-low · low-demand context

### P-high · high-demand context

A · Low-instability / non-ACL
B · Occult / associated
C · Compensated ACL-deficient
D · Overt ACL-instability
filled squares = F I R M unfavourable

## Anticipated objections

The strongest questions we expect — answered before you ask them. Disagreement is welcome: it is how this framework will be validated.

What exactly counts as «functional instability»?

**An instrumentally demonstrable dynamic control deficit of the injured lower limb** — deliberately broader than hop distance, and broader than the knee seen in isolation. It includes movement quality, loading symmetry, timing and limb coordination during gait, landing, deceleration, directional change and return-to-sport tasks. The focus shifts from the ACL as a structure to the **ACL-deficient limb as a dynamic system** [10, 11, 15, 29].

**How it is measured — three tiers.** Tier 1: laboratory biomechanics (3D motion capture, force plates, instrumented RTS batteries). Tier 2: clinic-based instrumented movement analysis — MAT / Green Room-type systems [13] and KneeKG-type knee kinesiography [7, 8, 16]. Tier 3: scalable surrogates — 2D video analysis, LESS-type assessments, wearables, hop tests interpreted with movement-quality criteria. Any tier can populate F; the requirement is that the deficit is **demonstrable and reproducible**, not merely narrated.

**What F is not.** It is not the patient's sensation of instability — that is domain **I**. It is not proof of structural insufficiency — F can be driven by AMI, weakness, proprioceptive or kinetic-chain deficits with an intact ACL. And it is never a stand-alone surgical trigger: an isolated unfavourable F calls for a better understanding of the limb, not for an operation.


Why doesn't M include the Lachman or the pivot shift?

**Begin with the biomechanics.** The ACL is the *primary* restraint to anterior tibial translation and only a *secondary* restraint to internal rotation. Even within the pivot shift itself, the first event of the manoeuvre is the anterior subluxation of the tibia — the rotational reduction follows. Anterior translation is therefore not a surrogate of ACL insufficiency: it is its **core mechanical expression**, and it is precisely what instrumented testing measures best.

**Then, the honest part: no test is operator-free.** Arthrometry and even stress radiography carry a human component — the purpose of M is to **minimise** it, not to pretend it is zero. Instrumented testing returns a *number on a continuous scale* with published reference standards [17] — documentable, comparable across centres and over time; properly executed stress radiography (Telos-type) is arguably the most reliable of these options. An awake pivot shift, by contrast, returns a *grade*, with well-known inter-rater variability and strong dependence on patient guarding.

Manual examination — Lachman, pivot shift — remains essential to clinical assessment and to the verification loop: it informs the clinician; it does not adjudicate the discriminating domain. The residual rotatory question is not lost either: R positivity without instrumented AP laxity classifies as phenotype **B (occult / associated)** and triggers targeted work-up, where anterolateral pathology belongs. Validated quantitative pivot-shift instrumentation — triaxial accelerometry, KiRA-type systems, developed and validated by the Bologna group at the Istituto Ortopedico Rizzoli [30, 31] — is a natural candidate input for M as validation matures, with the honest caveat that, among instrumented options, its operator-dependence is currently the highest.


Which patients is this for — revisions, children, multi-ligament injuries?

The framework was conceived around the **primary ACL injury in the skeletally mature knee**. Its formal boundaries — revisions, partial tears, multi-ligament patterns, the skeletally immature — are part of the agenda for the content-validation and consensus process. Until those boundaries are defined, clinical judgement governs the edges, as it should.


My findings are borderline, and my patient is neither low- nor high-demand.

Correct on both counts — and acknowledged in the publication itself. Conceptually the domains are **ordinal** (favourable / borderline / unfavourable) and patient context is a spectrum; this matrix is a deliberately **binary operational translation** for case-by-case use. Resolve borderline findings clinically before using the matrix, and treat the two P columns as poles, not boxes. Finer granularity is exactly what the validation process is designed to refine.

**The same honesty applies to numerical thresholds.** FIRM-P deliberately sets no universal cut-offs, because the literature itself has not agreed on them: for KT-1000 side-to-side difference some centres call ≥3 mm pathological, others ≥4 or ≥5 mm; for posterior tibial slope, proposed risk thresholds range from 12° to 14–15° depending on the series and the measurement method; LSI cut-offs of 85% and 90% coexist. Pretending one of these numbers is settled would be improvisation dressed up as precision. What the framework asks instead is operational: **declare which reference standard your centre uses, state it in the record, and apply it consistently** [17] — and when a value sits near your declared threshold, treat the domain as unresolved and re-measure. The verification loop exists precisely for borderline numbers.


I don't have force plates, arthrometry or stress radiography.

The reasoning sequence still applies — describe, verify, then contextualise — and the Functional domain is explicitly tiered down to clinic-level surrogates (2D video, quality-scored hop testing). One boundary must stay sharp: **those surrogates replace laboratory movement analysis within F only**. They measure movement quality, not laxity — and functional performance correlates poorly with instrumented laxity [1] [10], which is precisely why F and M are separate domains rather than substitutes for one another. No hop test and no video analysis can answer the M question. Where M cannot be measured, the honest position is that the pattern is **incomplete**, and the next step is obtaining the measurement — typically by referral to a centre that has it — not guessing it. A dedicated «incomplete data» mode for non-specialist settings — which tests to order, whom to refer to — is on the roadmap, together with additional languages and offline use.


Real measures are continuous. Why force a binary Yes/No on each domain?

Because the framework dichotomises at the **decision layer**, not at the **measurement layer**. Each domain answer is a clinical judgement informed by continuous measures interpreted against published reference standards [17] — the binary form is what makes the reasoning explicit, auditable and comparable between observers. When a measure sits near a threshold, the honest answer is not to hide the uncertainty inside a weighted score: it is to flag the domain as unresolved and re-measure — which is exactly what the verification loop does. Borderline values are a reason to verify, never a reason to guess.


Does this apply to every knee — children, multiligament injuries, revisions?

No, and the framework does not pretend to. FIRM-P addresses the primary decision problem in **ACL-related instability of the adult knee**. Skeletally immature patients, multiligament injuries, and revision settings carry additional decision drivers — growth plates, combined laxities, graft and tunnel factors — that sit outside the 32-scenario matrix. In those contexts FIRM-P can still structure the instability part of the reasoning, but it must not be read as the whole decision. Declaring the boundary of a tool is part of what makes it defensible.


You preach measurement — yet the framework itself is not validated.

Correct — and the distinction matters. FIRM-P is published as a **conceptual framework**: a reasoning structure, not a measurement instrument. What it asks of each domain is measurable today with validated tools; what is not yet demonstrated is the reliability of the framework as a whole. That work is the declared next step, not an afterthought: an inter-rater reliability study — surgeons and physiotherapists grading the same standardised cases — is in preparation, and substantive results will be submitted for peer review. Until then, the framework claims exactly what it can defend: that making the reasoning explicit is better than leaving it implicit. That is a claim about transparency, not about outcomes.


Hasn't this been done before — copers, IKDC, consensus statements?

Parts of it, yes — and the debt is acknowledged. The coper / non-coper literature demonstrated long ago that ACL-deficient knees are not one population; consensus work such as the Panther Symposium [6] and shared decision-making research [12] mapped the treatment question thoroughly. FIRM-P stands on that ground and adds four things those sources do not provide together: an **explicit, mutually exclusive classification rule**; the formal **separation of knee phenotype from patient context**; **AMI as a cross-domain modifier**; and a complete **operationalisation into 32 auditable scenarios**. The claim is not that the ingredients are new. The claim is that the architecture is.


This page is polished. How do I know the science underneath is solid?

Check it — the page is built to be checked. Every reference is verified against PubMed/Crossref and individually linked, so any citation can be audited in seconds. The classification rule is recomputed against all 32 scenarios **live in your browser** every time the page loads (the integrity badge above the map). Every scenario, every stance and every threshold statement is inspectable, and errors can be reported directly to the authors, who take full responsibility for the intellectual content. Polish is a courtesy to the reader; **verifiability is the guarantee**.


Is this a medical device? Could its output be used in a medico-legal dispute?

No — and it is deliberately designed not to be one. The simulator processes **no individual patient data**: it explores 32 predefined, hypothetical scenarios, the way an interactive textbook chapter would. Its outputs are stances for discussion — «offer and discuss», never «operate» — and applying the framework to a real patient requires the full assessment and the responsibility of the treating clinician. For the same reason, its output has no standing as an individual recommendation: it cannot «contradict» a clinical decision, because it never issues one. Should a future version accept individual patient data, it would first be reassessed under the applicable medical-device regulation.


Could this tool let non-physicians decide treatment?

No — and not because of a disclaimer, but because of the architecture. FIRM-P is **multi-professional in assessment and medical in decision**, and the two are not in tension: they are the design.

Look at the domains. F is where physiotherapy expertise is at its strongest — movement quality, loading, dynamic control — and the framework gives that expertise a formal, citable channel into the decision. But R requires the interpretation of MRI and radiographs, and M requires prescribing and interpreting stress radiography or instrumented laxity: in most jurisdictions these are **medical acts**. A FIRM assessment therefore cannot even be completed without a physician — the framework makes collaboration structural, not optional.

The decision itself — surgical or conservative — is a therapeutic indication, and it follows a principle that applies to every profession, physicians included: **decision-making authority and accountability are inseparable**. Whoever takes a treatment decision answers for it professionally, legally and financially. The treating physician carries that accountability in full, and the framework does not transfer it — it makes the reasoning behind it transparent, auditable and shared.

Read this way, the framework enlarges the physiotherapist's role rather than shrinking it: their findings stop being an informal opinion and become formal inputs the physician must reason with. **FIRM-P distributes the assessment. It does not distribute the prescription.**


Why is a scientific framework being communicated publicly?

Because dissemination is part of the work. A framework meant to change how clinicians reason is useless if only its authors know it exists. The public communication around FIRM-P is educational and citational — the model, the evidence, the paper — with a simple internal rule: facts, not promises; the framework, not the individuals. Open science includes explaining the work clearly outside the journal, to the professionals who will use it and the patients who will benefit from it.

## Authorship & open use

Clear ownership, open application. This framework exists for one beneficiary only: the patient.

**Conceptual authorship**

The FIRM-P framework — its domains, its classification rule, the verification loop and the 32-scenario matrix — is the intellectual work of **Dario GiunchiiD, Matteo DentiiD and Marc Barrera UsoiD**. Authorship of the concept remains with the authors in all uses and derivatives.

**Open clinical protocol**

FIRM-P is published to be used. Clinicians, physiotherapists, institutions and educators are **free to apply, teach and build upon** this framework wherever it benefits patients — provided the authors' work is cited (citation below).

**Validation is welcome**

Independent projects aiming to validate FIRM-P — content validity, inter-rater reliability, prospective derivation — are **permitted, encouraged and actively supported**. Write to the authors at studio.giunchi@hin.ch or via dariogiunchi.ch.

**Purpose**

The ultimate goal of this framework, of its matrix and of this simulator is to **help the patient — and no one else**. Any use consistent with that goal is in the spirit of FIRM-P.

**A living framework — version note**

FIRM-P evolves faster than journals print. The version you are using here is **already ahead of the version described in the original publication**: peer feedback is incorporated continuously, and the article is the frozen, citable snapshot. The concept, domains, rule and 32-scenario matrix are unchanged; what evolves is their operational refinement. Substantive evolutions of the model will themselves be submitted for peer review as an update. Cite the paper; use the simulator; if they differ in a detail, this page is the more recent statement of the authors' position.

Version history — latest: v1.12

**v1.12** — current. Manuscript accepted by J Exp Orthop: status updated to in press.  
**v1.11** — roles made explicit: assessment is multi-professional, the treatment decision is a medical act (new objection, stance note, footer).  
**v1.10** — clarified that field surrogates (video analysis, hop tests) belong to F only and never substitute for M.  
**v1.9** — anticipated objections expanded: validation status, lineage, verifiability, regulatory & medico-legal position, public communication.  
**v1.8** — FIRM-P wordmark trialled in the masthead, then reverted to the plain kicker.  
**v1.7** — full UI/UX audit; version history reordered newest-first.  
**v1.6** — objections on dichotomisation and scope moved into the Anticipated objections section.  
**v1.5** — cut-off heterogeneity made explicit (KT-1000 SSD, tibial slope, LSI); this version note added.  
**v1.4** — quantitative pivot-shift instrumentation credited and referenced [30, 31]; privacy statement.  
**v1.3** — biomechanical rationale for M expanded (anterior translation as primary restraint); section navigation.  
**v1.1–v1.2** — peer feedback integrated: primary Yes/No answers with classification tags, per-domain assessment checklists as aide-memoire, explicit "not a score" statement, operational definition of functional instability.  
**v1.0** — as submitted with the JEO manuscript: 4+1 domains, explicit rule, verification loop, 32-scenario matrix (v5).

**How to cite**

Giunchi D, Denti M, Barrera Uso M. FIRM-P separates knee phenotype from patient context in ACL-related instability: a conceptual framework integrating arthrogenic muscle inhibition as a cross-domain modifier. Journal of Experimental Orthopaedics (ESSKA). Accepted for publication (in press) — DOI will appear here upon publication.

Copy citation

Conflict of interest: the authors use movement-analysis systems of this class in routine clinical practice, but have no financial or commercial relationship with any manufacturer cited on this page.

## Evidence base

Every reference below was verified against PubMed/Crossref (authors, journal, year, DOI). Numbering 1–29 matches the manuscript's reference list; [30, 31] are simulator-specific additions on quantitative pivot-shift instrumentation.

**FIRM-P** — Giunchi DiD, Denti MiD, Barrera Uso MiD. FIRM-P separates knee phenotype from patient context in ACL-related instability. *Journal of Experimental Orthopaedics* (ESSKA), accepted for publication, in press. This simulator operationalises the publication's 32-scenario matrix.

Educational prototype for clinicians, exploring predefined hypothetical scenarios. Not a validated score, not a medical device, and not a substitute for clinical judgement. Treatment decisions — surgical or conservative — are medical acts and rest with the treating physician.

Runs entirely in your browser — no patient data is entered, stored or transmitted.

Spotted an error? Tell us — corrections make the framework better: studio.giunchi@hin.ch

**FIRM-P Simulator v1.12** · matrix v5 · August 2026
